# Structure-based screening of FDA-approved drugs identifies potential histone deacetylase 3 repurposed inhibitor: molecular docking and molecular dynamic simulation approaches

**DOI:** 10.3389/fphar.2024.1424175

**Published:** 2024-06-28

**Authors:** Anas Shamsi, Mohd Shahnawaz Khan, Dharmendra Kumar Yadav, Moyad Shahwan

**Affiliations:** ^1^ Center for Medical and Bio-Allied Health Sciences Research, Ajman University, Ajman, United Arab Emirates; ^2^ Department of Biochemistry, College of Science, King Saud University, Riyadh, Saudi Arabia; ^3^ Department of Pharmacy, College of Pharmacy, Gachon Institute of Pharmaceutical Science, Gachon University, Incheon, Republic of Korea; ^4^ Department of Clinical Sciences, College of Pharmacy and Health Sciences, Ajman University, Ajman, United Arab Emirates

**Keywords:** histone deacetylase 3, FDA approved drugs, imatinib, carpipramine, virtual screening, molecular dynamic (MD) simulations

## Abstract

Histone deacetylase 3 (HDAC3) is a member of the histone deacetylase family that has emerged as a crucial target in the quest for novel therapeutic interventions against various complex diseases, including cancer. The repositioning of FDA-approved drugs presents a promising avenue for the rapid discovery of potential HDAC3 inhibitors. In this study, we performed a structure-based virtual screening of FDA-approved drugs obtained from DrugBank. Candidate hits were selected based on their binding affinities and interactions with HDAC3. These promising hits were then subjected to a comprehensive assessment of their biological properties and drug profiles. Our investigation identified two FDA-approved drugs, Imatinib and Carpipramine, characterized by their exceptional affinity and specificity for the binding pocket of HDAC3. These molecules demonstrated a strong preference for HDAC3 binding site and formed interactions with functionally significant residues within the active site pocket. To gain deeper insights into the binding dynamics, structural stability, and interaction mechanisms, we performed molecular dynamics (MD) simulations spanning 300 nanoseconds (ns). The results of MD simulations indicated that Imatinib and Carpipramine stabilized the structure of HDAC3 and induced fewer conformational changes. Taken together, the findings from this study suggest that Imatinib and Carpipramine may offer significant therapeutic potential for treating complex diseases, especially cancer.

## 1 Introduction

Epigenetics has emerged as a critical field in understanding the regulation of gene expression and its role in health and disease (Zhang et al., 2020). One of the primary mechanisms in epigenetics is histone modification, which encompasses acetylation and deacetylation processes (Daskalaki et al., 2018). The addition and removal of acetyl groups on histone proteins are dynamically regulated by histone acetyltransferases (HATs) and histone deacetylases (HDACs), respectively (Lee and Grant, 2019). These modifications have a profound impact on chromatin structure, influencing the accessibility of DNA to transcription factors and RNA polymerase, thereby governing gene expression (Venkatesh and Workman, 2015). Histone deacetylase 3 (HDAC3) is a pivotal regulator of gene expression and chromatin structure that plays a central role in epigenetic modifications (Chen et al., 2015). It is crucially involved in various cellular processes, such as cell cycle control, differentiation, and apoptosis, underscores its significance in health and disease (Sarkar et al., 2020). Dysregulation of HDAC3 activity has been implicated in several complex diseases, making it an attractive target for therapeutic intervention (Sarkar et al., 2020). In recent years, there has been a surge of interest in developing HDAC3 inhibitors as potential therapeutic agents (Amin et al., 2019; Sarkar et al., 2020).

One notable area of application for HDAC3 inhibitors is anticancer therapy (Hu et al., 2019). However, achieving selectivity and specificity for HDAC3 over other HDAC isoforms is a critical challenge (Cheshmazar et al., 2022). The existence of multiple HDAC isoforms with distinct functions necessitates the design of molecules that target HDAC3 without affecting other isoforms, to avoid potential off-target effects. Various FDA-approved drugs, such as Imatinib and Carpipramine have great potential to be explored as repurposed molecules against HDAC3-associated diseases. Imatinib is a tyrosine kinase inhibitor initially developed for chronic myeloid leukemia (CML) and later extended to gastrointestinal stromal tumors (GIST) (Joensuu and Dimitrijevic, 2001; Demetri, 2002). It widely exhibits promising potential beyond its original indications that showcases efficacy in various therapeutic areas (Stegmeier et al., 2010). Carpipramine is a tricyclic antipsychotic primarily used for schizophrenia, and holds promise for repurposing in cancer treatment, although its application in this context remains largely unexplored (Kishi et al., 2014).

In the realm of drug discovery, the repositioning of existing FDA-approved drugs offers an attractive strategy for expediting the development of new therapies (Cha et al., 2018). This approach leverages the extensive knowledge of these molecules, including their safety profiles, pharmacokinetics, and mechanisms of action, to identify novel indications and therapeutic targets (Pushpakom et al., 2019). The advantages of drug repositioning are manifold (Hodos et al., 2016). Firstly, the drug development process is accelerated, as many aspects of preclinical and early clinical testing have already been conducted. Secondly, the safety and tolerability profiles of these drugs have been established, reducing the risk associated with novel molecules. Finally, the cost and resources required for repositioning are often significantly lower than those for *de novo* drug development (Cha et al., 2018). In the context of HDAC3 inhibition, the repositioning of FDA-approved drugs presents a rational and efficient approach. By screening these molecules for their potential to interact with HDAC3 and modulate its activity, we can identify promising candidates for further development as HDAC3 inhibitors. This approach aligns with the broader strategy of precision medicine, as it seeks to target specific molecular pathways associated with diseases while minimizing potential side effects.

The primary objective of this study is to perform structure-based virtual screening of FDA-approved drugs sourced from DrugBank (Wishart et al., 2018), with a focus on identifying molecules that can serve as potential HDAC3 inhibitors. We have employed a multi-step approach, including molecular docking and molecular dynamics (MD) simulations, to select and characterize the candidate molecules. Ultimately, we aim to identify and characterize FDA-approved drugs with the potential to serve as lead candidates for the development of HDAC3 inhibitors. These inhibitors might have the potential to play a pivotal role in the treatment of complex diseases, with a particular focus on cancer. The implications of HDAC3 inhibition extend beyond cancer therapy, encompassing a wide range of complex diseases. Epigenetic modifications have been implicated in neurodegenerative disorders (Kwon et al., 2016), metabolic syndromes (Carson and Lawson, 2018), and autoimmune diseases (Ramming et al., 2014), among others (Duthie, 2011). Overall, this study embarks on a journey to explore the potential of repositioning FDA-approved drugs as HDAC3 inhibitors, addressing the pressing need for novel therapeutic strategies in the battle against complex diseases, with a particular emphasis on cancer. The outcomes of this study may hold transformative implications for the treatment of complex diseases, opening doors to more precise and personalized therapeutic interventions.

## 2 Materials and methods

### 2.1 Computational tools and web servers

This study was executed on an HP^®^ Z840, a tower workstation configured for dual-boot functionality with Windows 11 and Ubuntu LTS, boasting 128 GB of RAM, 48 GB of dedicated GPU memory, and a spacious 2 TB SSD. Throughout the study, a reliable power source ensured uninterrupted operation, complemented by high-speed internet connectivity. A variety of bioinformatics software and online resources were employed for data analysis, molecular docking, visualization, and MD simulations. To perform molecular docking, we utilized well-established bioinformatics software, including MGL Tools (Huey et al., 2012), AutoDock Vina (Trott and Olson, 2010), and Discovery Studio (Biovia, 2017). These tools facilitated the reasonable prediction of ligand-receptor interactions. For the visualization and in-depth analysis of molecular structures and interactions, PyMOL (DeLano, 2002)and VMD: Visual Molecular Dynamics (Humphrey et al., 1996) were instrumental. These software tools allowed us to gain insights into the binding modes and structural dynamics of the complexes. Additionally, QtGrace (Turner, 2005) was employed for data visualization and presentation. GROMACS (Van Der Spoel et al., 2005) was employed to conduct all-atom simulations. This software enabled us to explore the conformational changes, stability, and interaction dynamics of HDAC3 in complexes with the identified molecules over an extended period. Several valuable online resources were consulted throughout the study. The Protein Data Bank served as a crucial source for retrieving the HDAC3 structure. Additionally, DrugBank played a pivotal role in providing essential drug-related information for the evaluation and analysis of the data.

### 2.2 Retrieval and processing of HDAC3 structure and FDA-approved drugs

The atomic coordinates for the crystal structure of HDAC3 (PDB ID: 4A69) (Watson et al., 2012) were sourced from the Protein Data Bank (Berman et al., 2000). In preparation for virtual screening, we removed all co-crystallized ligands and associated crystal water molecules from the parent structure. These co-crystallized ligands and associated crystal water molecules were removed to ensure that the binding affinity of the screened drugs was not influenced by pre-bound molecules. The resultant HDAC3 structure was refined and prepared for subsequent analysis. To ensure the protein reaches its most stable conformational state, the structure was subjected to energy minimization after ligand removal. To facilitate this process, MGL Tools, a versatile suite of software tools, was employed. Simultaneously, we obtained a diverse library of 3,648 FDA-approved drugs from DrugBank in the PDBQT file format. This library encompassed the three-dimensional representations of these pharmaceutical molecules, which were essential for our structure-based virtual screening analysis. The inclusion of the HDAC3 structure and this comprehensive library of FDA-approved drugs was fundamental to our investigation, enabling precise molecular docking and the subsequent evaluation of potential HDAC3 inhibitors among these molecules.

### 2.3 Molecular docking

Molecular docking approach was conducted to assess the binding affinity of molecules towards HDAC3. AutoDock Vina within the PyRx platform (Dallakyan and Olson, 2015) was used for the docking screening. This approach allowed us to evaluate the interactions between HDAC3 and the FDA-approved drugs. For the molecular docking procedure, a structurally blind approach was implemented, ensuring that all molecules were assessed without bias. The docking grid dimensions were set to 67, 67, and 73 Å, while the center coordinates for X, Y, and Z were specified as 42.751, 52.009, and 19.418, respectively. A grid spacing of 1 Å and an exhaustiveness parameter of eight were chosen to ensure thorough exploration of the binding conformations and to maximize the accuracy of the binding affinity predictions.

### 2.4 Selection of hits

The initial selection of potential hits was a meticulous process driven by their binding affinities and interactions with HDAC3. We prioritized molecules that exhibited higher binding affinities for HDAC3. This step allowed us to identify candidates with a strong potential to modulate HDAC3 activity. To comprehensively assess the interactions, all possible conformers of the selected molecules were generated. We performed this step using Discovery Studio, a versatile tool for molecular analysis and visualization. Subsequently, we conducted a detailed interaction analysis of all the docked conformers. This step enabled us to identify selective molecules that interacted effectively with the binding pocket of HDAC3. In the final stage of selection, we exclusively chose molecules that demonstrated interactions with functionally significant binding-site residues of HDAC3. This stringent criterion ensured that the selected hits had the potential to influence the critical regulatory aspects of HDAC3. Through this approach, we pinpointed a subset of molecules that exhibited strong binding affinities and engaged with functionally relevant residues in the HDAC3 binding site. These molecules emerged as the most promising candidates for further investigation and development as potential HDAC3 inhibitors.

### 2.5 Molecular dynamics simulations

All-atom MD simulations play a crucial role in drug discovery, particularly in understanding the dynamic behavior of proteins and their interactions with ligands (Shukla and Tripathi, 2020). For an in-depth exploration of the protein and protein-ligand complexes, MD simulations were conducted. These simulations spanned a duration of 300 ns and involved both the free-HDAC3 and HDAC3 complexed with the identified molecules, Imatinib and Carpipramine. The simulations were performed at a constant temperature of 300 K with the charmm36-jul2022 force field (Huang and MacKerell, 2013). GROMACS version 5.1.2 served as the primary software tool for this purpose. All systems, including free-HDAC3 and HDAC3 in complex with Imatinib and Carpipramine, were immersed in a cubic box of water with dimensions of 10 Å. The solvation was achieved using the TIP3P model (Mark and Nilsson, 2001) through the *gmx solvate* module. Prior to the simulations, an energy minimization step was executed to remove unfavorable interactions. This involved 1,500 steps of steepest descent energy minimization to optimize the system’s stability. A controlled temperature ramp-up was implemented during the equilibration phase, transitioning the system from 0 K to the target temperature of 300 K. This was performed over a 1 ns period, maintaining constant volume and utilizing periodic boundary conditions. Post-simulation, the resulting trajectories were analyzed using various utilities available within GROMACS (Van Der Spoel et al., 2005). These analyses allowed us to gain a comprehensive understanding of the system’s behavior and interactions. All graphs and figures, illustrating the outcomes of the MD simulations, were prepared using QtGrace, enhancing the clarity and presentation of the results. A comprehensive and detailed description of the MD simulation procedures and parameters can be found in our recent publications (Mohammad et al., 2020; Shamsi et al., 2021).

## 3 Result and discussion

### 3.1 Molecular docking: binding affinity with HDAC3

Molecular docking plays a crucial role in understanding the interactions between drugs and their target proteins at the molecular level (Singh et al., 2022). Here, we refined the results from an initial pool of 3,648 molecules, strategically narrowing down the selection to the top 10 hits. We have selected the top 10 hits from the pool of 3,648 molecules based on their binding affinity. This results in the identification of compounds with binding affinity within the range of −8.3 kcal/mol to −9.5 kcal/mol (Table 1). Notably, all these selected FDA-approved drugs exhibited a binding affinity surpassing that of the reference HDAC3 inhibitor, RGFP966 (Zhang et al., 2021). RGFP966 is a widely explored compound that acts as a selective inhibitor of HDAC3 (Leus et al., 2016; Zhang et al., 2021; Sun et al., 2022). This finding strongly indicates the potential of these hit molecules as promising candidates for the development of HDAC3 inhibitors. The superior binding affinity displayed by these molecules underscores their efficacy in interacting with HDAC3, suggesting a high likelihood of successful inhibition. This outcome holds significant promise for advancing our understanding of HDAC3 inhibition and provides a foundation for further exploration and development of novel therapeutic interventions.

**TABLE 1:** Selected hits and their docking score with HDAC3.

S. No.	Drug	Binding Energy (kcal/mol)	Ligand Efficiency (kcal/mol/non-H atom)	Torsional Energy
1	Picloxydine	−9.5	0.2969	0.6226
2	Aprepitant	−9.0	0.2432	2.4904
3	Conivaptan	−8.9	0.2342	1.2452
4	Eltrombopag	−8.7	0.2636	2.1791
5	Dutasteride	−8.6	0.2324	1.2452
6	Penfluridol	−8.6	0.2389	2.8017
7	Deptropine	−8.5	0.34	0.6226
8	Imatinib	−8.5	0.2297	2.1791
9	Tadalafil	−8.4	0.2897	0.3113
10	Carpipramine	−8.3	0.2515	1.8678
11	RGFP966	−7.5	0.2778	2.1791

### 3.2 Interaction analysis with HDAC3

**FIGURE 1:**
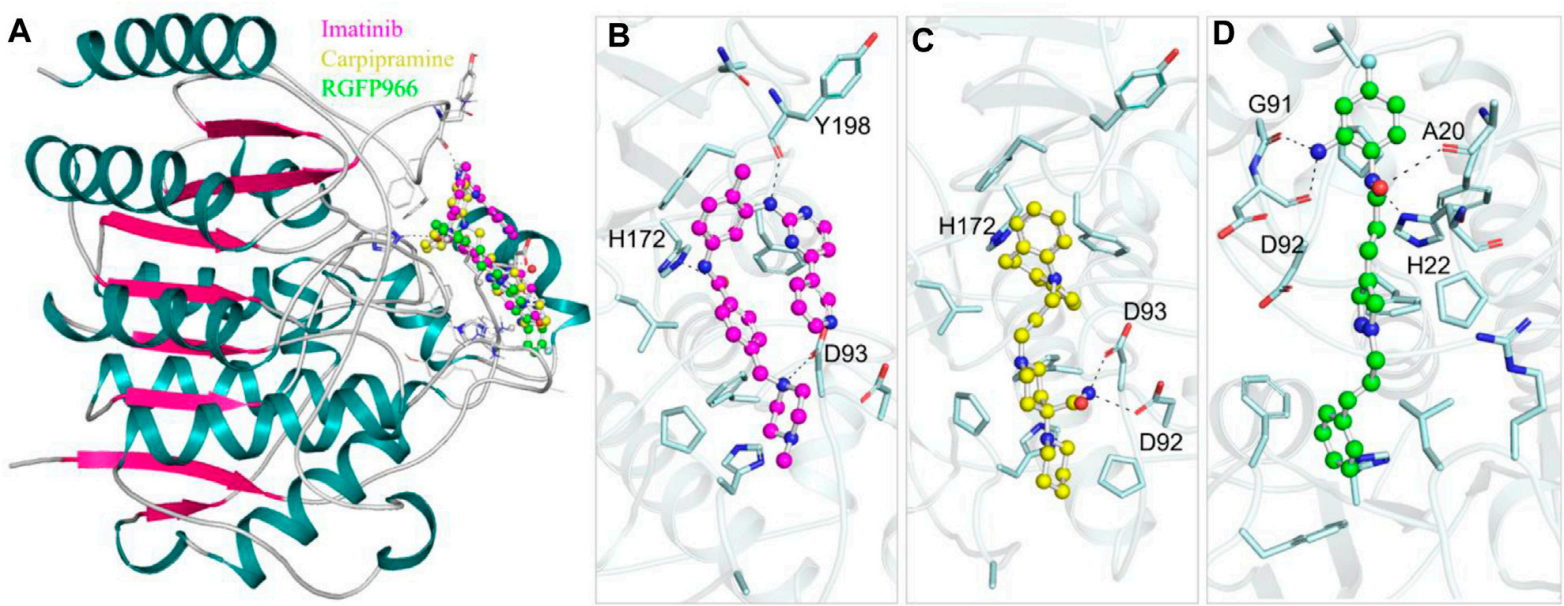
Protein-ligand interactions. **(A)** Cartoon representation showing the docked molecules in the binding pocket of HDAC3. **(B)** Imatinib, **(C)** Carpipramine, and **(D)** RGFP966 are shown in magnified view of HDAC3 binding site residues. Residues with polar interactions are labeled in black.

Protein-ligand interaction analysis is a critical component of drug discovery that provides valuable insights into the molecular mechanisms underlying the efficacy and specificity of potential drug candidates (Du et al., 2016). To gain a comprehensive understanding of the interactions between selected 10 molecules and HDAC3, we conducted a thorough analysis employing Discovery Studio. A total of 90 docked conformers, derived from the output files of these 10 molecules, were generated for analysis. Each conformer was examined for its interactions with HDAC3. During this analysis, we observed that several docked conformations of these molecules exhibited direct and specific interactions within the binding pocket of HDAC3. These interactions were of particular interest, as they indicated the potential of these molecules to modulate the activity of HDAC3. Following the analysis, we have identified two FDA-approved drugs, Imatinib and Carpipramine, which consistently exhibited significant interactions with crucial binding-site residues of HDAC3 (Figure 1). Both molecules were observed to bind in close proximity to the active site residue His135 of the binding site, displaying shared interactions with the reference inhibitor RGFP966 (Figure 1A). Notably, these molecules formed multiple hydrogen bonds with essential residues within the binding pocket (Figure 1B–D). HDACs are metalloenzymes that require metal ions, such as Zn^2+^, for catalytic activity (Nechay et al., 2016). Their metal-binding sites are crucial for guiding inhibitor design to achieve improved isozyme selectivity over promiscuous metal-chelating agents. Imatinib and Carpipramine bind at the metal-binding site on HDAC3, where the co-crystallized Zn^2+^ is located (Figure 1B, C) (Watson et al., 2012). Importantly, all three molecules demonstrated a commendable complementarity fit while effectively obstructing the binding site. The insights gained from this comprehensive interaction analysis shed light on the potential mechanisms through which Imatinib and Carpipramine may impact the functionality of HDAC3. This reinforces their viability as promising candidates for HDAC3 inhibitors, underlining their potential therapeutic significance.

**FIGURE 2:**
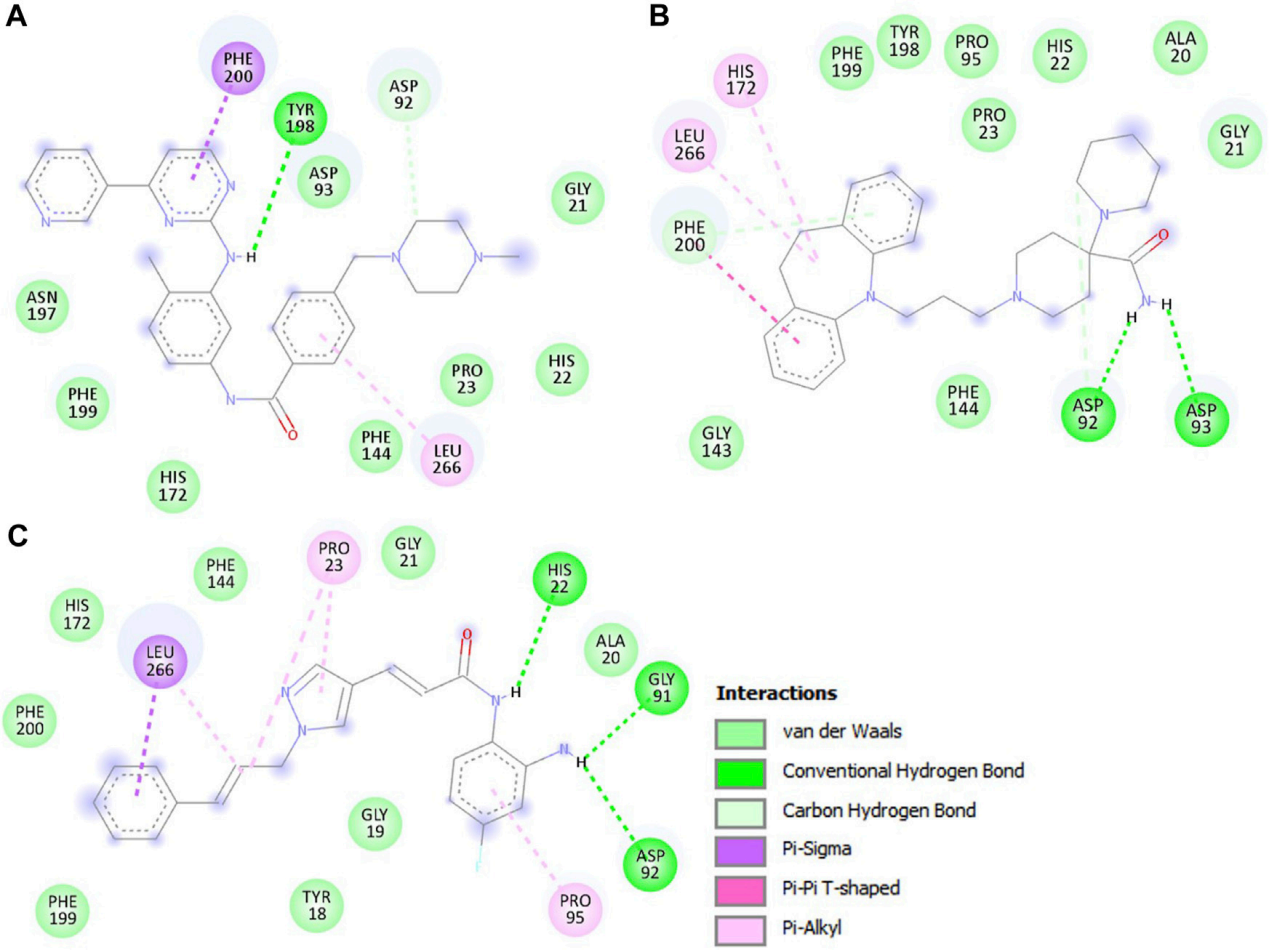
The detailed interactions between HDAC3 and the investigated molecules. **(A)** 2D structural representation HDAC3 residues interacting with Imatinib, **(B)** Carpipramine, and, **(C)** RGFP966.

Further, we conducted an in-depth analysis of the protein-ligand interactions between HDAC3 and the Imatinib and Carpipramine. The examination focused on their interactions with crucial binding-site residues of HDAC3, with particular attention to their comparison with the reference inhibitor RGFP966. Both Imatinib and Carpipramine demonstrated substantial interactions with key binding-site residues of HDAC3, suggesting a potential for therapeutic relevance. Notably, both molecules exhibited shared interactions with the reference inhibitor RGFP966, indicating their ability to engage the target site in a manner similar to the established inhibitor (Figure 2). Moreover, both molecules formed multiple hydrogen bonds and other interactions with essential residues within the binding pocket, highlighting their strong binding affinity. Specifically, Imatinib displayed a noteworthy interaction with Asp92 and Tyr198, forming two hydrogen bonds (Figure 2A). On the other hand, Carpipramine exhibited an even more extensive interaction profile, establishing four hydrogen bonds with Asp92, Asp93, and Phe200 (Figure 2B). When comparing the interactions of Imatinib and Carpipramine with the reference inhibitor RGFP966, it was observed that both molecules shared a common hydrogen bond with Asp92 (Figure 2C). RGFP966 formed three hydrogen bonds with His22, Gly91, and Asp92, emphasizing its crucial role in the binding pocket. The shared interaction with Asp92 between the investigated molecules and the reference inhibitor is particularly significant in the context of drug discovery projects. This suggests that both Imatinib and Carpipramine possess a binding profile that aligns with the established inhibitor, potentially making them promising candidates for further development.

### 3.3 Molecular dynamics simulations

#### 3.3.1 Structural deviations and compactness

**FIGURE 3:**
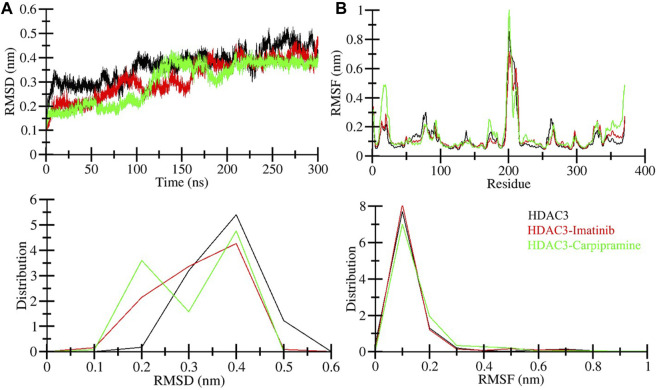
Structural dynamics of HDAC3 upon Imatinib and Carpipramine binding: **(A)** RMSD of HDAC3 in complex with Imatinib and Carpipramine. **(B)** Residual fluctuations (RMSF) of HDAC3 and upon Imatinib and Carpipramine binding. Black, red and green represent values obtained for free HDAC3, HDAC3-Imatinib and HDAC3-Carpipramine complexes, respectively.

The binding of small molecules within a protein’s binding pocket can induce significant conformational changes (Mobley and Dill, 2009). One of the fundamental metrics used to assess these structural deviations and the overall stability of a protein is the root mean square deviation (RMSD) (Maruyama et al., 2023). In the simulation analysis, we observed average RMSD values for three distinct systems: free HDAC3, HDAC3-Imatinib, and HDAC3-Carpipramine complexes, which were found to be 0.37, 0.32, and 0.30 nm, respectively. A noteworthy observation in the RMSD plot is a higher RMSD for free HDAC3 beyond the 120 ns mark. This deviation can be attributed to the increased flexibility of the loop of the HDAC3 structure. The RMSD plot provided valuable insights, demonstrating that the binding of both Imatinib and Carpipramine significantly stabilized HDAC3, resulting in fewer structural deviations from its native conformation (Figure 3A). Although some minor fluctuations were noted in the RMSD plot upon the binding of Imatinib and Carpipramine, these can be attributed to potential changes in the orientation of the molecules within the HDAC3 binding pocket. However, the binding of Imatinib and Carpipramine displayed consistently lower RMSD values in several regions, indicating equilibration throughout the simulation and the stability of HDAC3 (Figure 3A). This pattern can also be observed in the probability distribution function of the data (Figure 3A, lower panel).

To gain further insights into the local structural flexibility, we examined the root-mean-square fluctuation (RMSF) of individual residues in HDAC3 both in its native form and upon binding with Imatinib and Carpipramine. The RMSF plot revealed residual fluctuations in several regions of the protein structure. However, the binding of Imatinib and Carpipramine led to a noticeable reduction in these residual fluctuations throughout the simulation, particularly in the region spanning from the N-terminal to the C-terminal (Figure 3B). These observations suggest that the binding of Imatinib and Carpipramine stabilizes the overall structure of HDAC3 and minimizes local structural fluctuations, particularly in regions crucial for HDAC3 function. This enhanced structural stability is indicative of the potential therapeutic efficacy of these molecules as HDAC3 binders. To further support this observation, the probability distribution function of the data was examined (Figure 3B, lower panel). Consistent with the RMSF analysis, this distribution function corroborates the trend of reduced structural fluctuations in the presence of Imatinib and Carpipramine, providing additional evidence for the stabilizing effect of these molecules on HDAC3.

**FIGURE 4:**
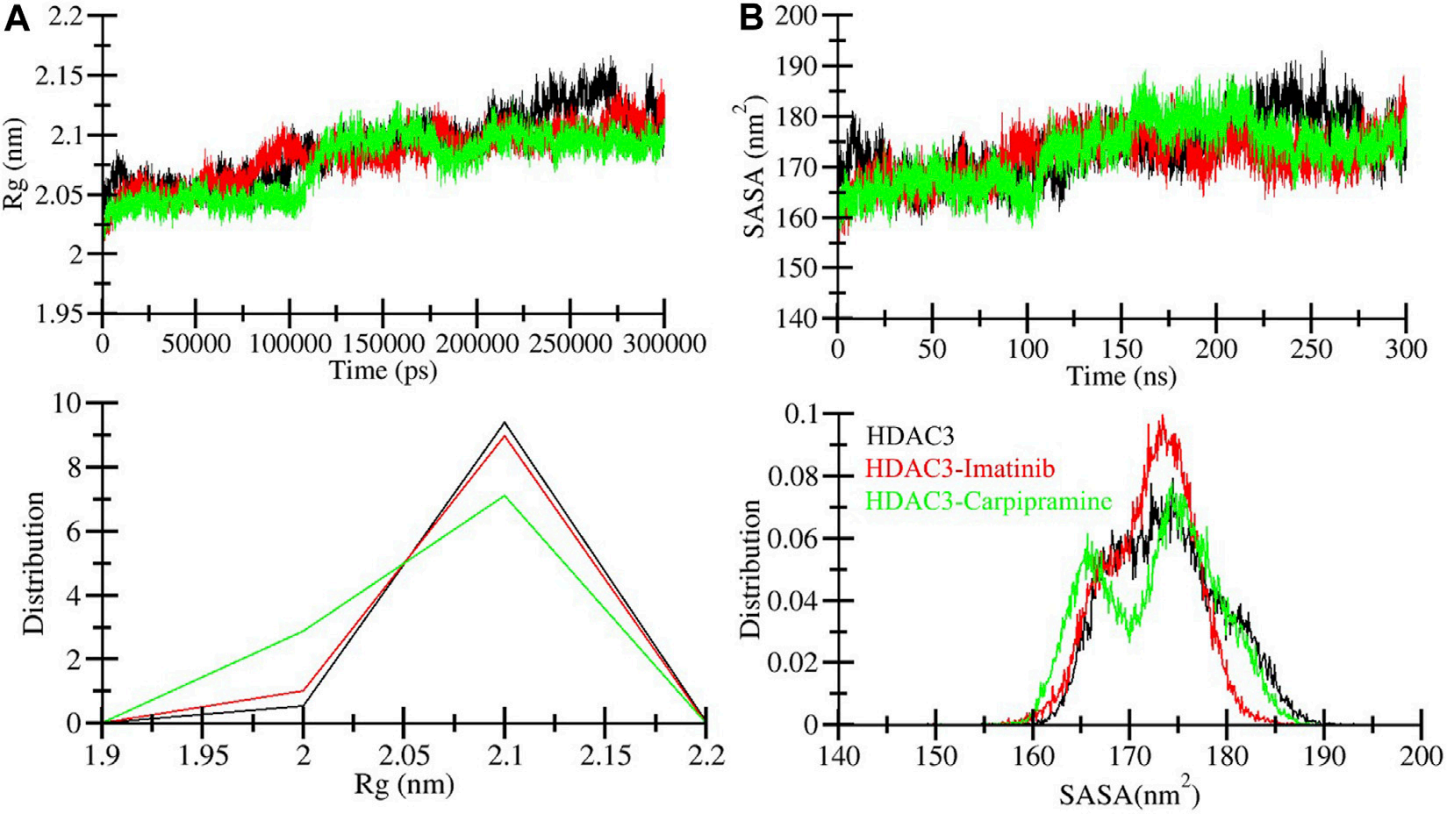
Compactness of HDAC3 upon Imatinib and Carpipramine binding: **(A)** Time evolution of radius of gyration (*Rg*). **(B)** SASA plot of HDAC3.

The radius of gyration (*R*g) is a crucial metric directly associated with the tertiary structure volume and overall conformational shape of a protein (Lobanov et al., 2008). It provides valuable insights into the stability of a protein, with a higher *R*g indicating less tight packing. We calculated the average *R*g values for three systems: free HDAC3, HDAC3-Imatinib, and HDAC3-Carpipramine complexes. These values were determined to be 2.09, 2.08, and 2.07 nm, respectively. The *R*g plot revealed no major changes in the packing of HDAC3 in the presence of Imatinib and Carpipramine. While some slight fluctuations were observed after the 40 ns mark in the MD trajectories, the *R*g plot reached a stable equilibrium throughout the 300 ns simulation (Figure 4A). The consistency in the *R*g plot indicated minimal structural deviation in HDAC3 upon the binding of Imatinib and Carpipramine. To further support this observation, the probability distribution function of the data was examined (Figure 4A, lower panel). Consistent with the *R*g plot, the probability distribution function highlighted the reliability of the data, emphasizing the minimal structural deviation in HDAC3 when bound to Imatinib and Carpipramine.

The solvent accessible surface area (SASA) corresponds to the surface area of a protein that interacts with its environment (Marsh and Teichmann, 2011). We calculated the average SASA values for free HDAC3, HDAC3-Imatinib, and HDAC3-Carpipramine complexes throughout the 300 ns MD simulation. The calculated average SASA values were 173.80, 172.06, and 172.68 nm^2^ for free HDAC3, HDAC3-Imatinib, and HDAC3-Carpipramine, respectively. Notably, there was a slight increment in SASA values, potentially due to the tighter packing of HDAC3 upon binding with Imatinib and Carpipramine (Figure 4B). This observation suggests that the binding of these molecules influenced the interactions between HDAC3 and its surrounding solvent. The modest change in SASA further supports the notion of enhanced stability and compactness of the HDAC3 structure when in complex with Imatinib and Carpipramine. Overall, the *R*g and SASA analyses reaffirm the structural stability and minimal conformational shifts within HDAC3 upon binding with Imatinib and Carpipramine, further highlighting their potential as HDAC3 inhibitors. To reinforce this observation, we analyzed the probability distribution function of the data (Figure 4B, lower panel). The congruence between the probability distribution function and the SASA plot underscores the trustworthiness of the data, highlighting the negligible structural deviation in HDAC3 when interacting with Imatinib and Carpipramine.

#### 3.3.2 Dynamics of hydrogen bonds

**FIGURE 5:**
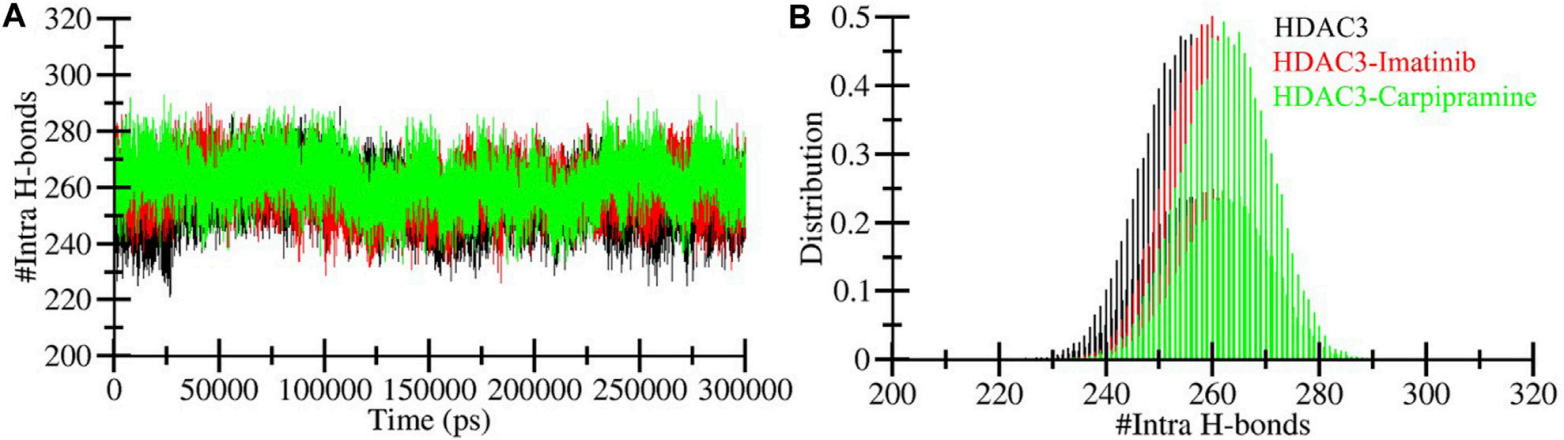
Time evolution of hydrogen bonds. **(A)** Intra-HDAC3 hydrogen bonds in HDAC3 and **(B)** their PDF values.

Intramolecular hydrogen bonding plays a pivotal role in maintaining protein stability (Yunta, 2017). The analysis of intramolecular hydrogen bonds can provide valuable insights into the overall stability of protein structures. Additionally, intermolecular hydrogen bond analysis allows us to examine the polar interactions between a protein and a ligand, shedding light on the directionality and specificity of these interactions, a fundamental aspect of molecular recognition (Menéndez et al., 2016). In our pursuit to validate and assess the stability of HDAC3 in complex with Imatinib and Carpipramine, we delved into the dynamics of hydrogen bonds formed within a 0.35 nm distance during the simulation (Figure 5A). During the simulation, we calculated an average of hydrogen bonds within HDAC3 itself, revealing the following values: 255 for free HDAC3, 259 for HDAC3-Imatinib, and 262 for HDAC3-Carpipramine complexes (Figure 5B). These findings illuminate the stability of intramolecular hydrogen bonds within HDAC3 when it is complexed with Imatinib and Carpipramine.

**FIGURE 6:**
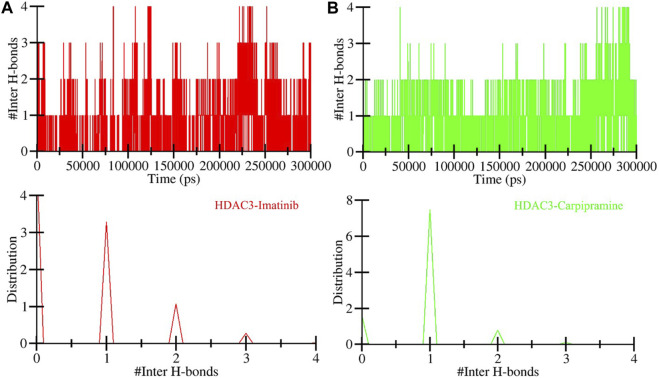
Time evolution of hydrogen bonds. **(A)** Intermolecular hydrogen bonds between Imatinib and HDAC3, and **(B)** Intermolecular hydrogen bonds formed between Carpipramine and HDAC3.

In the context of intermolecular calculations, we focused on the number of hydrogen bonds formed between Imatinib and HDAC3, as well as Carpipramine and HDAC3. Throughout the simulation, an average of 2 hydrogen bonds were consistently observed in both the Imatinib-HDAC3 and Carpipramine-HDAC3 interactions. This analysis provides direct evidence of the specific interactions occurring between the molecules and HDAC3. The dynamics of hydrogen bonds further revealed that Imatinib and Carpipramine bind within the active pocket of HDAC3. During this interaction, they form between 3 and 4 hydrogen bonds with higher fluctuations and between 1 and 2 hydrogen bonds with more minor fluctuations (Figure 6). This observation aligns closely with our molecular docking findings, reinforcing the notion of strong and specific interactions between Imatinib and Carpipramine with HDAC3 (Figure 6A, B). The analysis of hydrogen bonds underscores the structural stability of HDAC3 and emphasizes the robust and specific binding of Imatinib and Carpipramine to the active site of HDAC3, further solidifying their potential as effective HDAC3 binders.

#### 3.3.3 Secondary structure dynamics

**FIGURE 7:**
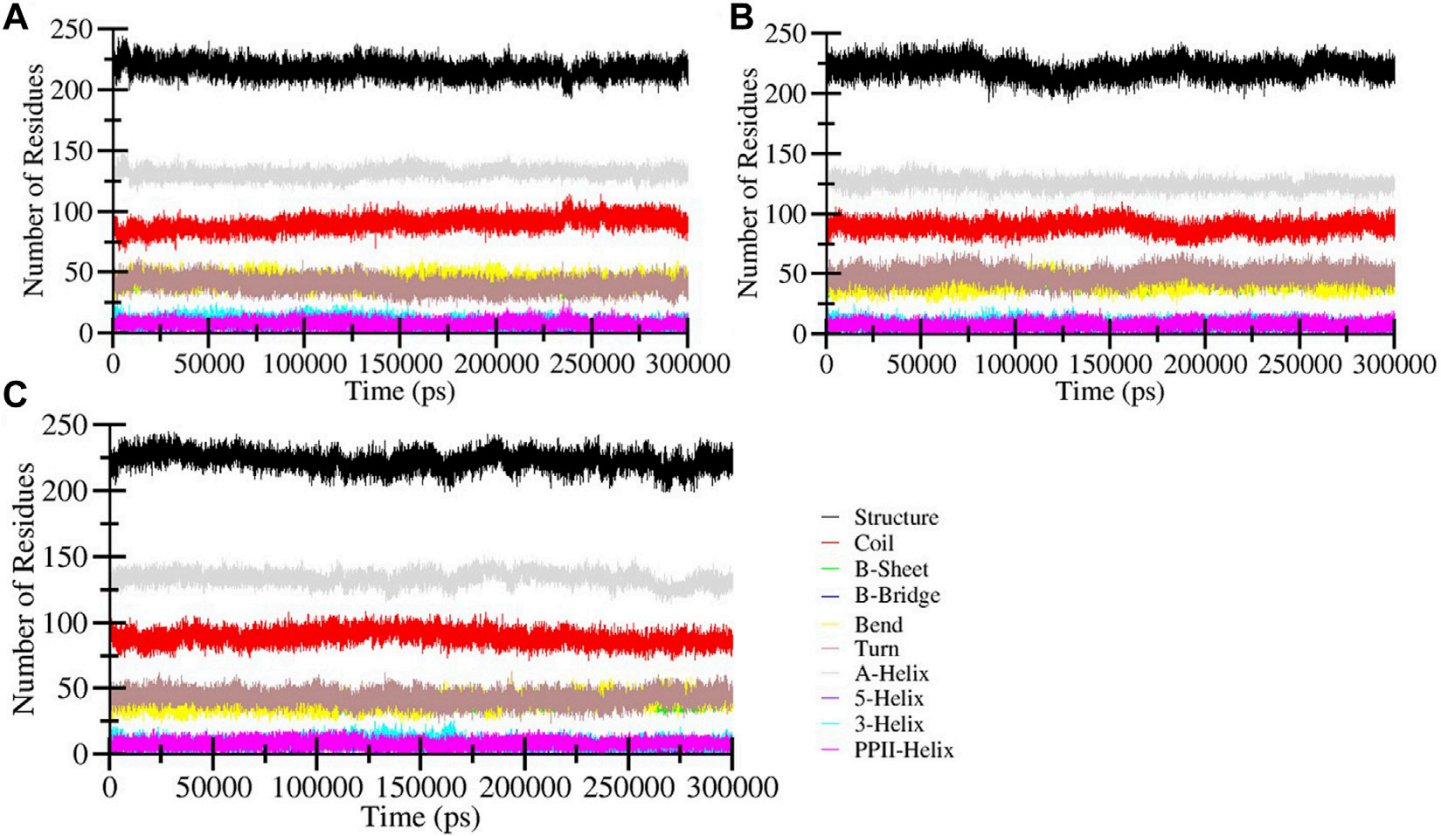
Secondary structure content of **(A)** Free HDAC3 and **(B)** HDAC3 upon Imatinib **(C)** Carpipramine binding. *Structure = α-helix + β-sheet + β-bridge + Turn.

To assess changes in the secondary structure of HDAC3 resulting from the binding of Imatinib and Carpipramine during the simulation, we calculated the structural content of HDAC3. This analysis focused on key secondary structure elements such as α-helix, β-sheet, and turn. For each time step, we quantified the average number of residues participating in the formation of these secondary structure elements and plotted these values over time. The findings revealed that the secondary structure elements in HDAC3 remained remarkably constant and equilibrated throughout the entire simulation (Figure 7). No significant alterations were observed in the secondary structure content of HDAC3 upon the binding of Imatinib and Carpipramine (Figure 7B, C). This observation underlines the robust stability of the complexes and suggests that the structural integrity of HDAC3 is well-maintained during the interaction with these molecules. The preservation of secondary structure elements and minimal changes in their content further support the hypothesis that Imatinib and Carpipramine form stable complexes with HDAC3, emphasizing their potential as promising HDAC3 inhibitors.

### 3.4 Principal component analysis

**FIGURE 8:**
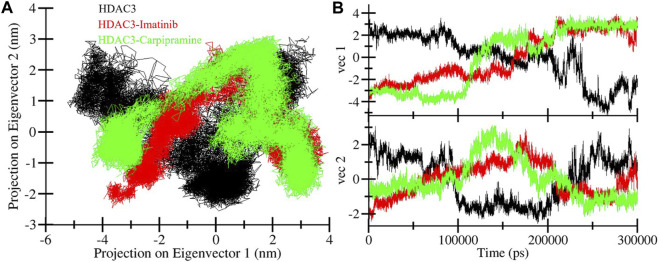
Principal component analysis. **(A)** 2D projections of trajectories showed different projections of HDAC3. **(B)** The time-evolution of the projection.

Principal component analysis (PCA) serves as a valuable tool to explore the global motion of a protein, reducing this complexity to a few principal motions characterized by eigenvectors (Muller Stein et al., 2006). Here, we employed PCA to gain insights into the conformational dynamics of free HDAC3, as well as HDAC3 in complex with Imatinib and Carpipramine. The essential subspace of these complexes was analyzed, providing a visualization of the tertiary conformations along the first and second eigenvectors projected by the Cα atom (Figure 8). The analysis revealed that HDAC3 explores a broad range of phase spaces in the presence of Imatinib and Carpipramine, highlighting a cluster of stable states (Figure 8A). Importantly, this observation underscores that the protein maintains its overall conformational stability and that no substantial shifts or switching in the global motion of HDAC3 were observed after the binding of Imatinib and Carpipramine (Figure 8B). These findings indicate that the binding of Imatinib and Carpipramine does not induce significant changes in the overall motion of HDAC3. The protein retains its stability while accommodating the presence of these molecules, which aligns with the earlier observations of structural and interaction stability.

### 3.5 Free energy landscape analysis

**FIGURE 9:**
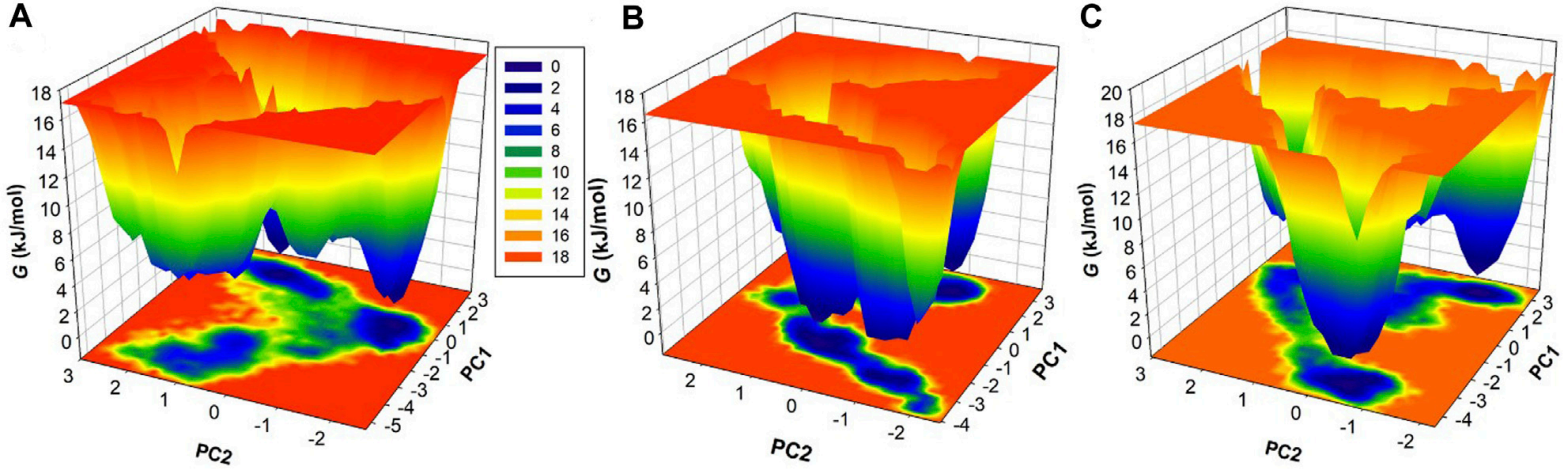
The free energy landscapes obtained for **(A)** HDAC3, **(B)** HDAC3-Imatinib, and **(C)** HDAC3-Carpipramine.

To delve deeper into the conformational behavior of the systems, we conducted an analysis of the Gibbs free energy landscapes (FELs) using the first two eigenvectors (Figure 9). The FELs provide critical insights into the energy landscapes of the different systems (Papaleo et al., 2009). As depicted in Figure 9A, the FEL of free HDAC3 revealed multiple stable global minima, primarily confined within 3 basins. However, a striking change in conformational behavior was observed when HDAC3 was in the presence of Imatinib and Carpipramine (Figure 9B, C). The FELs for these complexes displayed a progression to multiple energy minima, signifying the acquisition of numerous stable states by HDAC3. These multiple energy minima suggest a stable and diverse conformational landscape in the presence of Imatinib and Carpipramine, indicative of the impact of these molecules on the conformational folding of HDAC3. The alteration in the FELs emphasizes the considerable influence of Imatinib and Carpipramine on the conformational dynamics of HDAC3, further underlining their potential as modulators of HDAC3 behavior.

## 4 Conclusion

In this study, we embarked on a comprehensive exploration of the potential for repurposing FDA-approved drugs as inhibitors of HDAC3. We employed a systematic approach that integrated molecular docking, MD simulations, and structural analyses and identified two repurposed drugs, Imatinib and Carpipramine, targeting HDAC3. The analysis of binding affinities through molecular docking pinpointed Imatinib and Carpipramine as potent candidates with remarkable affinities towards HDAC3. Subsequent MD simulations revealed that the binding of these molecules substantially stabilized the HDAC3 structure, leading to fewer structural deviations from its native conformation. The analysis of hydrogen bonding dynamics unveiled strong, specific interactions between Imatinib and Carpipramine with functionally crucial residues of HDAC3. This validates our molecular docking findings and supports the role of these molecules as potential HDAC3 inhibitors. PCA elucidated the global motion of HDAC3, revealing that it maintains stability while accommodating the presence of Imatinib and Carpipramine. The FEL analysis underscored the dynamic conformational behavior of HDAC3 in the presence of these molecules, suggesting a more diverse landscape of stable states. In summary, our study provides compelling evidence for the potential of Imatinib and Carpipramine as effective HDAC3 repurposed inhibitors. These molecules exhibit strong binding affinities and stabilize the structural and dynamic behavior of HDAC3. Their ability to maintain the protein’s structural integrity and preserve specific interactions highlights their promise for therapeutic applications against a spectrum of complex diseases, such as cancer. The findings of this research contribute to the growing body of knowledge surrounding drug repurposing and emphasize the potential of FDA-approved drugs as valuable candidates for the development of novel therapeutic agents targeting HDAC3.

## Data Availability

The original contributions presented in the study are included in the article/Supplementary material, further inquiries can be directed to the corresponding author.
